# Cognitive modelling of concepts in the mental lexicon with multilayer networks: Insights, advancements, and future challenges

**DOI:** 10.3758/s13423-024-02473-9

**Published:** 2024-03-04

**Authors:** Massimo Stella, Salvatore Citraro, Giulio Rossetti, Daniele Marinazzo, Yoed N. Kenett, Michael S. Vitevitch

**Affiliations:** 1 CogNosco Lab, Department of Psychology and Cognitive Science, University of Trento, Trento, Italy; 2 Institute of Information Science and Technologies, National Research Council, Pisa, Italy; 3 Faculty of Psychology and Educational Sciences, Department of Data Analysis, University of Ghent, Ghent, Belgium; 4 Faculty of Data and Decision Sciences, Technion - Israel Institute of Technology, Haifa, Israel; 5 Department of Speech Language Hearing, University of Kansas, Lawrence, KS USA

**Keywords:** Cognitive modelling, Multilayer networks, Multiplex networks, Cognition, Knowledge modelling, Cognitive data science

## Abstract

The mental lexicon is a complex cognitive system representing information about the words/concepts that one knows. Over decades psychological experiments have shown that conceptual associations across multiple, interactive cognitive levels can greatly influence word acquisition, storage, and processing. How can semantic, phonological, syntactic, and other types of conceptual associations be mapped within a coherent mathematical framework to study how the mental lexicon works? Here we review cognitive multilayer networks as a promising quantitative and interpretative framework for investigating the mental lexicon. Cognitive multilayer networks can map multiple types of information at once, thus capturing how different layers of associations might co-exist within the mental lexicon and influence cognitive processing. This review starts with a gentle introduction to the structure and formalism of multilayer networks. We then discuss quantitative mechanisms of psychological phenomena that could not be observed in single-layer networks and were only unveiled by combining multiple layers of the lexicon: (i) multiplex viability highlights language kernels and facilitative effects of knowledge processing in healthy and clinical populations; (ii) multilayer community detection enables contextual meaning reconstruction depending on psycholinguistic features; (iii) layer analysis can mediate latent interactions of mediation, suppression, and facilitation for lexical access. By outlining novel quantitative perspectives where multilayer networks can shed light on cognitive knowledge representations, including in next-generation brain/mind models, we discuss key limitations and promising directions for cutting-edge future research.

## Introduction

The mental lexicon is a complex system where knowledge of the words and concepts one knows can be represented as units that are combined and associated across multiple levels (Aitchison, 2012). For example, phonemes combine to form words, words combined in sentences express ideas, and sentences in narratives give rise to stories (Litovsky et al., 2022; Vitevitch, 2019). Focusing on the level of units of words (which provide meaning even in isolation), deeper knowledge can be expressed by linking together units that are associated in some way. Words can be associated in many ways (Aitchison, 2012; Fillmore, 2006; Zock & Biemann, 2020). For example, words may share meaning (Steyvers & Tenenbaum, 2005), sound similar (Vitevitch, 2008), be syntactically related (Semeraro et al., 2022), bring other words to mind (De Deyne et al., 2013), represent objects with similar semantic or visual features (Kennington & Schlangen, 2015), be written similarly (Siew & Vitevitch, 2019), or evoke the same set of emotions and affective states (Mohammad & Turney, 2013). These are only some of the many ways in which words can be associated (Aitchison, 2012; Baronchelli et al., 2013; Stella, 2020; Vitevitch, 2019) and give structure to the knowledge that one has that can be expressed through language.

Decades of research in psycholinguistics and cognitive science have examined how the words and concepts in the mental lexicon are acquired, stored, processed, and retrieved (Aitchison, 2012; Collins & Loftus, 1975; Kumar, 2021; Kumar et al., 2022; Zock & Biemann, 2020). Importantly, it has been shown that the structure and organization of the words and concepts associated in some way in the mental lexicon influence a wide variety of linguistic and cognitive phenomena, such as word confusability (Vitevitch, 2019), picture naming (Castro & Siew, 2020; Castro & Stella, 2019; Castro et al., 2020), and memory recall patterns for both neutral (Kenett et al., 2017; Kumar et al., 2020) and emotional information (Dover & Moore, 2020; Fatima et al., 2021).

The structure and organization of the words and concepts associated in some way in the mental lexicon can be influenced by various factors, including psychedelic drugs (Rastelli et al., 2022), and how creative (Kenett & Faust, 2019; Kenett et al., 2018), expert (Koponen, 2021) or curious (Zurn & Bassett, 2018) an individual is. All these findings converge on one point: Understanding the structure and organization of knowledge in the mental lexicon is important for shedding light on a number of phenomena. Understanding the structure and organization of knowledge in the mental lexicon requires a framework that is quantitative (Siew et al., 2019), interpretable (Rudin, 2019), and human-centric (Bryson & Theodorou, 2019). This framework must: (i) be capable of producing inferences and comparable measurements regulated by mathematical equations and theoretical models (Castro & Siew, 2020; Christensen & Kenett, 2023; Zemla et al., 2020) (quantitative); (ii) map results to outputs through an internal representation of knowledge available to researchers, unlike most black-box machine-learning knowledge models (Fatima et al., 2021) (interpretable); and (iii) be grounded in psychological theory and large-scale datasets in order to account for the complex nuances of human psychology rather than make abstract inferences that are of little value to psychologists (Benedek et al., 2023; Wulff et al., 2019; Zaharchuk & Karuza, 2021). An artificial intelligence that categorizes individuals using binary labels like “aphasic” or “healthy” without identifying the severity of their language impairments, nor considers their ability to acquire, retain, and produce new knowledge would not be human-centric (Castro et al., 2020).

In the present review, we advance the idea of using multilayer networks to model and understand the structure and organization of knowledge in the mental lexicon. We review recent cutting-edge advancements and insights obtained in psychology by adopting the quantitative framework of multilayer networks, specifically in terms of: (i) detecting the presence and structure of a language kernel within the cognitive reflection of language in the mind, i.e., the mental lexicon, and assessing the impact of such a kernel for enhanced language acquisition in toddlers, children, and teenagers (Citraro et al., 2023; Stella et al., 2017; Stella et al., 2018); (ii) multiplex network structure unveiling different recall strategies from semantic memory among individuals characterized by different clinical impairments in the aphasia spectrum (Baker et al., 2023; Castro & Stella, 2019; Castro et al., 2020), different creativity levels (Stella & Kenett, 2019) and high/low levels of Openness to Experience (Samuel et al., 2023); (iii) multiplex networks identifying the presence of an interplay/interaction mechanism between semantic relatedness and phonological similarities in the mental lexicon, whereas combining these types of conceptual associations gives rise to shortcuts between semantic themes (clusters of concepts with similar meanings) and phonological communities (clusters of concepts with similar sounds) (Citraro & Rossetti, 2020; Levy et al., 2021).

Across all these research directions, multilayer networks can conveniently provide structure to conceptual similarities, without the need for resorting to a predetermined underlying vectorial space (e.g., in the Global Vectors for Word Representation model, GloVe, one would need to fix a priori the dimensions of vectors representing words, an arbitrary choice that makes it difficult to assess cognitive differences between vector representations of words in a 50-dimensional or a in 300-dimensional vectorial space). The main advantage of multilayer networks is their ability to provide different layers of structured associations between the same or different sets of nodes/entities (e.g., combine semantic and phonological associations between concept forms). This advantage translates into the necessity of carefully selecting these layers, possibly by following cognitive theories and models (Aitchison, 2012; Dóczi, 2019) from decades of research in cognitive science and psychology.

This review outlines how a careful selection of semantic, syntactic, and phonological layers, previously tested in separation when modelling the mental lexicon (Castro & Siew, 2020; Siew et al., 2019), can both unveil and reconstruct phenomena of relevance from a cognitive perspective, such as language spurts in toddlers (Citraro et al., 2023; Stella, 2019; Stella et al., 2017), influence mechanisms between personality traits, creativity, and recall in fluency tasks (Samuel et al., 2023; Stella & Kenett, 2019), mechanisms of failing word retrieval in people affected by disorders from the aphasia spectrum (Baker et al., 2023; Castro & Stella, 2019), and also glean insights into the structural organization of conceptual similarities in-between phonological and semantic aspects of words (Citraro & Rossetti, 2020; Levy et al., 2021). This review outlines, discusses, and organizes such findings in view of relevant research about the mental lexicon and language processing (Dóczi, 2019; Hills & Kenett, 2022).

Whereas other recent approaches in quantitative psychology have started using single-layer networks to model individual aspects of the mental lexicon (Hills & Kenett, 2022; Kumar et al., 2022; Siew et al., 2019), multilayer networks represent a more general framework, a new direction that is importantly capable of encompassing in interpretable ways multiple aspects of language processing in a single model, a key requirement for addressing decades of cutting-edge psychology research pointing out how the mental lexicon and language processing work in highly multidimensional and complex ways (Bock, 1996; Dell et al., 2014; Vukić et al., 2020). This review thus appeals to cognitive and psychology researchers trying to learn more about how multilayer networks might be a powerful framework for modelling their multidimensional research data or for gaining more quantitative insights into the structure and organization of the mental lexicon across multiple layers.

Noticeably, the insights provided by multilayer networks are different from other important results obtained from neural networks or other computational approaches in computational psychology (Dell et al., 1997; McClelland & Elman, 1986; Rosenblatt, 1958) in that the network structure in multilayer networks provides direct, interpretable access to the organization of conceptual associations without requiring training data or iterative updates of model weights. We further discuss this difference in the next section.

### Cognitive multilayer networks differ from artificial neural networks

Networks are a convenient and powerful way to study and visualize complex systems made of multiple interacting units (Berlingerio et al., 2011; De Domenico, 2022). Starting from graph theory, a subfield of mathematics dealing with units (called nodes) and links between them (called edges), network science has evolved as its own “applied” branch (Berlingerio et al., 2011; Newman, 2010). Network science, for example, studies social networks, where nodes are people and links are social interactions (Freeman, 2004), or transportion networks, where nodes are places and links are connections between them (Newman, 2010), and so on. Similarly, in “neural networks” the units are “neurons” or information-processing units, and the links are the information being transferred (Piloto et al., 2022).

In psychology, another type of “network” that has been seen in numerous eras, including present-day deep-learning networks (Piloto et al., 2022), is the artificial neural network (ANN), also sometimes called a connectionist model or parallel distributed processing (PDP) model (see the works by Rosenblatt (1958), Grossberg (1972), and Rumelhart et al. (1986)). These mathematical models, inspired by the way that neurons in the brain work, are often implemented as computer programs to simulate some sort of cognitive processes (e.g., Rumelhart et al., 1986). In these networks, nodes represent idealized or simplified neurons that can be arranged in layers. Nodes/artificial neurons from one layer can be linked to others in other layers. As opposed to cognitive networks, in ANNs, each node can “fire” and pass on activation to nodes in another layer, for example, multilayer perceptrons (Gardner & Dorling, 1998).

Although many of the above ANN models have multiple layers in the network, the nodes in these models are better described as processing units rather than representational units. In ANNs, information is represented by the patterns of activation that are distributed across a layer of nodes (with different patterns representing different concepts). In contrast, the multilayer networks we describe in this review use individual nodes to represent pieces of information (such as individual phonemes, words, etc.), with links connecting nodes if those pieces of information are related in some way (Levy et al., 2021; Stella, 2018). Thus, these multilayer networks form a map of different types of knowledge (Hills & Kenett, 2022; Vukić et al., 2020).

Cognitive processing can be modelled on such networks by a random walk or the diffusion of “activation” across the network (Abbott et al., 2015; Chan & Vitevitch, 2009; Kenett & Austerweil, 2016; Siew, 2019; Vitevitch & Mullin, 2021), but without endowing nodes with any ANN-like processing power. A central tenet of network science – the structure of the network influences cognitive processing (Siew et al., 2019; Vitevitch & Mullin, 2021) – further distinguishes the multilayer networks we describe here from ANN models of the mental lexicon (e.g., the TRACE model of speech perception; McClelland & Elman, 1986). Although both are “multilayer networks” and are used to model the mental lexicon, artificial multilayer neural networks focus on brain-inspired distributed processing of knowledge (Gardner & Dorling, 1998; McClelland & Elman, 1986; Rumelhart et al., 1986), whereas cognitive multilayer networks reconstruct and represent the structure of associative knowledge. We will use “multilayer network” in the latter connotation for the remainder of this review.

### Review outline

We discuss recent work from multiple fields to show how multilayer networks are a quantitative, interpretable, and human-centric framework that can connect several disparate disciplines interested in modelling knowledge. Multilayer networks are a cutting-edge approach to explore how knowledge is processed simultaneously across multiple levels. We outline three recent research developments where the ability to combine different layers of associative knowledge highlights phenomena that would be otherwise lost in single-layer network analyses or through other modelling approaches like word embeddings (Rudin, 2019). We discuss key limitations of this framework and review potential approaches for future research in cognitive modelling (Hills & Kenett, 2022; Siew et al., 2019; Vitevitch & Mullin, 2021) and cognitive neuroscience (Poeppel & Idsardi, 2022; Zaharchuk & Karuza, 2021). Combining evidence from fields as diverse yet interconnected as cognitive psychology, complexity science, and computer science, our review identifies concrete innovative ways in which multilayer networks can advance our understanding of cognition.

## Evidence for the multilayered nature of the mental lexicon

Despite the name, the mental lexicon is not a simple dictionary (Dóczi, 2019; Hills & Kenett, 2022; Kumar, 2021; Kumar et al., 2022; Vitevitch, 2019; Zock & Biemann, 2020). Concepts in the mental lexicon are not recalled in alphabetical order and the recollection of an item is not independent of other concepts associated with it (Kenett et al., 2017; Kumar et al., 2020). Aitchison (2012) used the London tube train system as a metaphor for the mental lexicon, where stations represent linguistic units and are connected according to a layout of channels of different lengths. This analogy resonates with the concept of a complex network, although the exact specification of structure, function, and dynamics in the mental lexicon is more sophisticated (Dóczi, 2019). Even though the mental lexicon might not be a network itself, some of its associative features might be accurately modelled by network science (Hills & Kenett, 2022).

Many research findings indicate that information represented in the mental lexicon is inherently multi-layered: Phonological, semantic, and syntactic aspects of language can simultaneously interact and influence language retrieval and processing (Castro & Siew, 2020; Dell et al., 1997; Zock & Biemann, 2020). In healthy populations, the interaction of multiple types of linguistic interactions in the mental lexicon is highlighted by the phenomenon of the tip of the tongue (Zock & Biemann, 2020), where an individual is aware of the semantic features of a word but cannot produce it. This tip-of-the-tongue state is characterized by a failure to retrieve phonological information, whereas semantic activation seems to be intact (Brown, 1991; Peng et al., 2022). Another example of faulty retrieval is known as a *malapropism* (Fay & Cutler, 1977; Peng et al., 2022), where a similar sounding word is retrieved for another semantically appropriate one (e.g., “dancing a flamingo” instead of “dancing a flamenco”). The faulty interaction between semantic and phonological information of words can also explain the increase of mixed errors in people with aphasia in a picture-naming task (Dell et al., 1997).

Evidence for the multilayered nature of the mental lexicon also comes from facilitative effects in word production like priming (Aitchison, 2012; Kenett et al., 2017; Kumar et al., 2020), i.e., when lexical retrieval is facilitated by cues related to target words. Morphological content (e.g., “dog” containing phonemes \d\, \o\and \g\), synonym similarities (e.g. “character” and “nature” being synonyms), and syntactic relationships (e.g., being a certain part of speech) were found to facilitate lexical retrieval through priming indicating the simultaneous interplay between phonological, semantic, and syntactic layers of the mental lexicon (Bock, 1996; Dóczi, 2019).

These findings motivated the formulation of the so-called cognitive linguistic theory (García Mayo et al., 2013), of serial lexical access (Dell et al., 1997) and of cobweb theory (Aitchison, 2012), which all argue that language production depends on a network of interacting layers of the mental lexicon, including individual phonemes, word meaning, and sentence structuring. Given the interaction of various types of information in the lexicon, the framework of multilayer networks becomes a natural way of analyzing the structural and dynamical complexity of the mental lexicon.

## From single-layer to multilayer networks as models of cognition

Complex networks represent the structure of pairwise connections between interacting entities (Newman, 2018). Connections are usually called links or edges, and the interacting entities are usually called nodes or vertices. A single-layer complex network represents only one type of relationship between nodes. Instead, both “multiplex” and “multilayer” networks include multiple types of relationships between nodes (Boccaletti et al., 2014).

For the sake of an easier visualization and to fully characterize the mathematics of such multilayer/multiplex networks, usually nodes are organized in sub-groups called network layers (De Domenico et al., 2015; Santoro & Nicosia, 2020), which identify specific aspects of the pairwise interactions between individual nodes. A single network layer is composed of a specific type of interaction between nodes (Stella et al., 2017). Links connecting nodes from the same network layer are called intra-layer links. Links connecting any two nodes from different network layers are called inter-layer links (Boccaletti et al., 2014).

Whereas single-layer networks can account only for one type of associative link, for example, syntactic relationships (Corrêa et al., 2020), the main advantage of multilayer networks is the ability to combine multiple types of associations within a single model (Battiston et al., 2020; Boccaletti et al., 2014; De Domenico et al., 2013; Kivelä et al., 2014). The presence of multiple aspects or layers of associations can give rise to phenomena greatly different from single-layer networks, such as the presence of feedback loops across layers (Kovács et al., 2021; Kumar et al., 2020), changes in the centrality of individual nodes when multiple interactions are simultaneously present (Bianconi, 2018), or the emergence of patterns of connectedness undetectable in the individual layers (Battiston et al., 2020; Battiston et al., 2014).

Both “multilayer” and “multiplex” networks have multiple network layers and represent multiple types of interactions, but these two terms are not synonyms (Bianconi, 2018; De Domenico et al., 2013; Kivelä et al., 2014). Multilayer networks represent a more general category of complex networks, whereas multiplex networks are a more specific network model because they feature *node alignment* (Battiston et al., 2020). Node alignment means that the same set of nodes are found in every network layer, with the same node being connected across layers. The presence of explicitly weighted intra-layer connections characterizes full multiplex networks. Although Collins and Loftus (1975) discussed the idea of multilayer phonological/syntactic networks of conceptual associations in the 1970s, Cong and Liu (2014) were the first to implement a multilayer representation of language representing syntactic and phonological relationships between Mandarin words. Importantly, Martinčić-Ipšić and colleagues (2016) extended the multilayer formalism to Croatian and English, highlighting several structural similarities between the two languages, which differ in terms of the allocation of syllables across words.

**Fig. 1:**
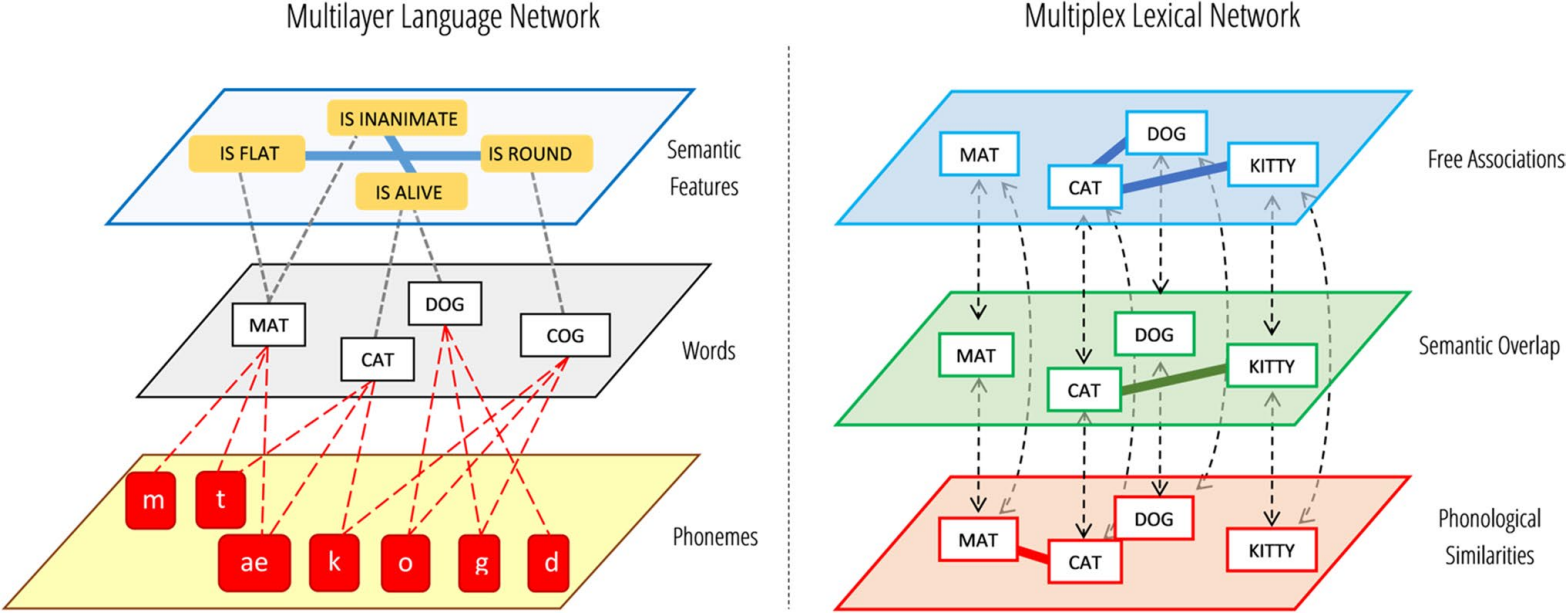
Examples of a multilayer language network (**left**; Cong & Liu, 2014) and of a multiplex network (**right**; Stella et al., 2017). If inter-layer connections were all unweighted, the multiplex network would be an edge-colored graph with three colors (free associations, semantic overlap, and phonological similarities). The multilayer language network maps semantic features and phoneme occurrence in words. Node alignment, the replication of the same set of nodes across layers, is a feature characterizing multiplex networks

Without node alignment, multilayer networks can feature different sets of nodes across layers, see Fig. 1 (left). One layer might feature phonemes, linked to a layer of words by several interlayer links expressing how phonemes occur in words. Words might also be linked to another layer expressing their semantic features, the latter being connected by intra-layer links expressing antonyms. Featuring different nodes across different layers makes the mathematics describing multilayer networks considerably more advanced than the mathematics behind multiplex networks (Bianconi, 2018; Boccaletti et al., 2014). Whereas a multiplex network can be described with matrices, multilayer networks require the use of tensors to represent them (for more details, see De Domenico et al., 2013; Kivelä et al., 2014).

Multilayer networks featuring node alignment are called multiplex networks [80]. Node alignment requires the same set of nodes to be replicated across all layers of a multiplex network (see also Fig. 1, right, where alignment is represented by dashed lines). Even if a node is disconnected on one layer of a multiplex network, but highly connected on another layer, the node has to appear in both layers. All the replicas of a given node represent different aspects of the same entity; for example, a phonological word form engaging in phonological relationships on a given layer and a lexical representation of the same word involved in conceptual associations on another layer (Stella, 2020; Stella et al., 2018). Replica nodes identify the so-called physical node, for example, in the above example the phonological form and the lexical representation are replicas identifying the same word/physical node (Battiston et al., 2014; De Domenico et al., 2013; Kivelä et al., 2014). Figure 1 provides an example of a multilayer language network, analogous to pioneering work by Cong and Liu (2014), and a multiplex lexical network, analogous to pioneering work by Stella and colleagues (2017).

**Fig. 2:**
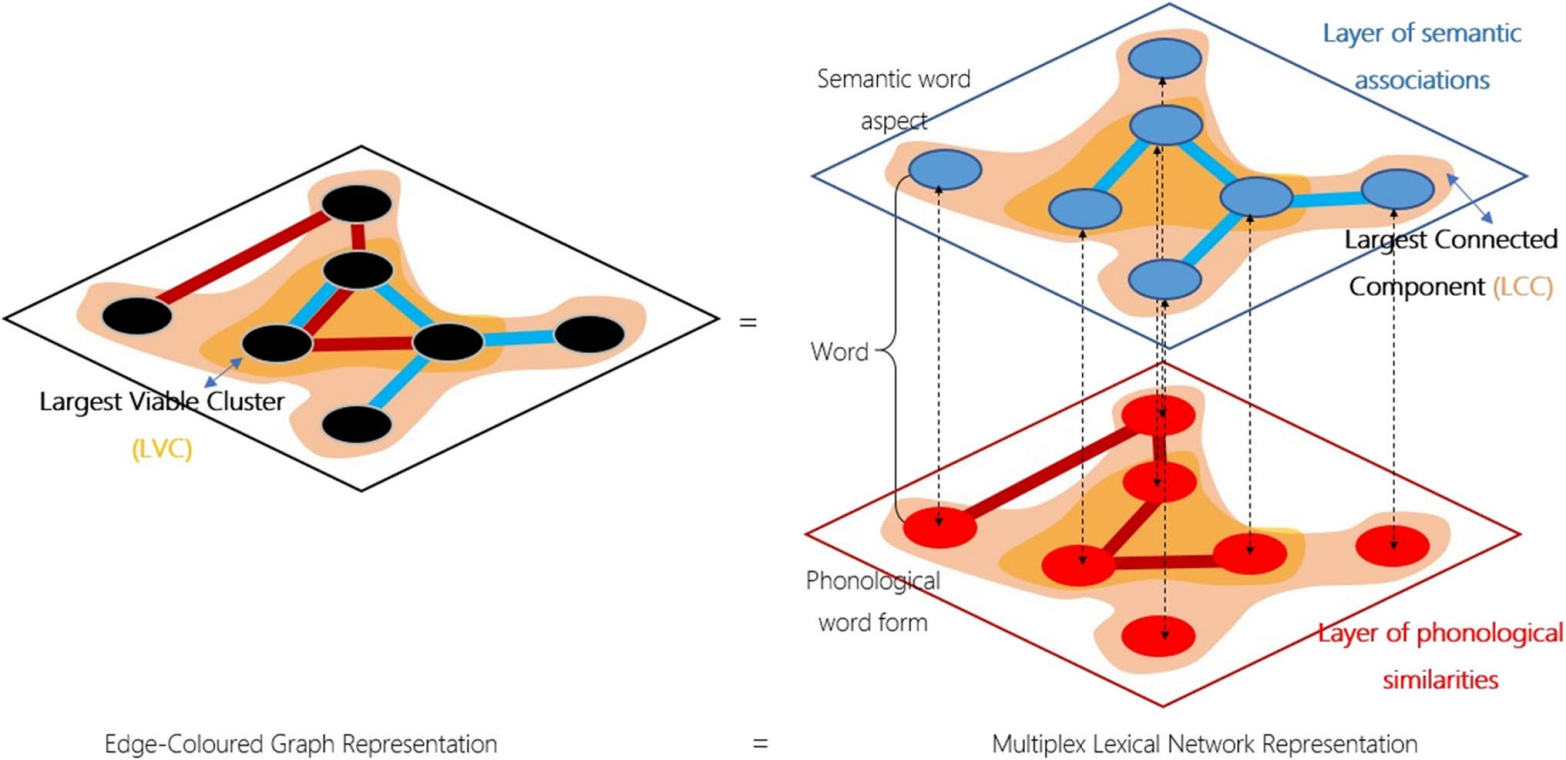
Edge-colored graphs are equivalent to multiplex lexical networks. Coloring edges according to their layer/nature can be convenient when one needs to account for connectivity from multiple sources combined (**left**). Distinguishing between layers (**right**) can be convenient when one wants to check for connectivity patterns on individual aspects of the mental lexicon, e.g. phonological similarities and semantic associations. Either representation captures the same connectivity patterns, one in terms of multidimensional colors for links and the other in terms of multiple layers. Notice that in multiplex lexical networks, connectivity can be described in two distinct ways: either in terms of connected components or in terms of viable clusters. The largest connected component (LCC, pink) is the largest set of nodes connected by at least one path with links of any color. The largest viable cluster (LVC, orange) is the largest set of nodes connected simultaneously on all layers by at least one path with links of the same color. The LVC is a more restrictive version of the LCC but the two coincide in single-layer networks

The layered structure of multilayer and multiplex networks enables the possibility of including both semantic and phonological features of linguistic units. For instance, Fig. 1, left, identifies semantic features and phoneme occurrences of words (Martinčić-Ipšić et al., 2016), whereas Fig. 1, right, maps semantic overlap, phonological similarities, and free associations between words (Stella et al., 2018). Notice that in multiplex networks it is only the type of interactions among nodes that changes across layers, whereas multilayer networks allow for a more flexible structure with the possibility of different types of nodes across layers. Notice also that in the absence of explicitly weighted intra-layer links, multiplex networks become edge-colored graphs (Battiston et al., 2020; Semeraro et al., 2022), where connections of different types are colored differently. In other words, edge-colored graphs are multiplex networks where links of different colors can be present at the same time between any two nodes, for example, phonological similarities and semantic associations, as highlighted in Fig. 2, left. A given color identifies a given type of relationships or similarity between any two nodes, i.e., all links of the same color can compose a given network layer, as highlighted in Fig. 2, right.

Multiplex lexical networks can be represented either by using the representation of edge-colored graphs or by using multiple layers, i.e., the representation of a multilayer network. The presence of multiple layers/interactions alters the connectivity of words drastically compared to their single-layer layouts (Cong & Liu, 2014; Martinčić-Ipšić et al., 2016). For instance, in Fig. 1, right, “mat” is disconnected on the free association layer but connected to “cat” on the phonological layer. Multiple layers also give rise to edge overlap across different aspects of associative knowledge; for example, “kitty” and “cat” share a connection on both the free association and the semantic overlap layers, an overlap that cannot be measured when layers are considered as separate, single components (De Domenico, 2022). In the following, we review how multilayer/multiplex networks can identify and quantify cognitive patterns that would go undetected using standard single-layer networks.

## Cognitive patterns highlighted by multilayer networks but invisible to single-layer networks

Multilayer networks take into account more than one type of relationship among nodes at once, thus giving rise to more complex structures and phenomena that cannot be observed in single-layer networks. This section reviews key ways in which multilayer networks have been shown to differ from single-layer networks. We discuss these quantitative differences in relation to the relevant psychology literature, and consider the overall benefits, limitations, and roads for future research of multilayer networks as models of cognition.

### Multiplex viability highlights language kernels in the mental lexicon invisible to single-layer networks

As in single-layer networks (Siew et al., 2019), the collection of all intra-layer and inter-layer links connecting nodes *i* and *j* also represents a path in multilayer networks (Bianconi, 2018). For instance, in Fig. 1, right, there is a multilayer path connecting “mat” and “kitty” through the intra-layer link “mat (phonological)” – “cat (phonological)”, the interlayer link “cat (phonological)” – “cat (semantic overlap)” and the intra-layer connection “cat (semantic overlap)” – “kitty (semantic overlap)”. In contrast to describing a path between nodes in a single-layer network, in multilayer paths it is necessary to specify the layers of nodes to distinguish intra- and inter-layer links. In edge-colored multiplex networks, this distinction is not necessary, but links of different colors must be allocated to different layers, i.e., one color identifies one layer (De Domenico, 2022; Vukić et al., 2020).

Notice that we say “edge-colored multiplex networks” because not all multiplex networks can also be represented as an edge-colored graph: Multiplex networks where inter-layer connections are present and with different weights cannot be cast into edge-colored graphs unless self-connections are considered. Assuming specific weights for inter- and intra-layer links enables the definition of the shortest path length, i.e., the minimum total weight or number of links necessary for traversing a path between any two nodes. In edge-colored graphs, the shortest path length (also called network distance or geodesic network distance) considers only intra-layer links, and can be defined as the smallest number of links of any color connecting any two nodes (Fatima et al., 2021). Without considering explicit inter-layer connections, the network distance between “mat” and “kitty” would be 2 in Fig. 1, left.

Ultimately, the possibility of “jumping” across layers enhances the connectivity of multilayer networks. Multilayer shortest path lengths in edge-colored multiplex networks mixing semantic, phonological, and syntactic associations were shown to significantly predict cognitive phenomena like early word acquisition (Citraro et al., 2023; Stella, 2019; Stella et al., 2017; Stella & De Domenico, 2018), semantic relatedness (Levy et al., 2021), and picture-naming production in people with aphasia (Castro & Stella, 2019; Castro et al., 2020). All these studies combined behavioral data in the form of free associations (Coltheart, 1981; De Deyne et al., 2019; De Deyne et al., 2013; Nelson et al., 2004) and linguistic data from WordNet (Miller, 1995) in the forms of synonyms, hypernyms, and phonological similarities (cf. Vitevitch, 2008). In all of these studies, multiplex network distances achieved better model performances than their single-layer counterparts i.e., in terms of achieving better** R**^2^ values and thus explaining more variance in the observed scores of semantic relatedness (Levy et al., 2021; Stella, 2018) and correct picture naming probabilities (Castro & Stella, 2019; Castro et al., 2020), but also in terms of multiplex network distances predicting normative word learning consistently better than single-layer distances or word frequency (Stella, 2019; Stella et al., 2017). These quantitative comparisons provide converging evidence that the ability to transition between layers, crucially enabled by multilayer networks, is pivotal for modelling interactive aspects of the mental lexicon that might intervene during mental navigation for memory search, retrieval, and recall (for a cognitive review of these processes, see Dell et al., 2014; Dell & O'Seaghdha, 1992; Hills et al., 2012; Hills & Kenett, 2022; Zock & Biemann, 2020) and during language acquisition (Dóczi, 2019; Kuperman et al., 2012; Vitevitch et al., 2023).

Interactions between different aspects of the lexicon might be modelled through explicit (Levy et al., 2021; Stella, 2018) or latent (Marinazzo et al., 2022) patterns of connectivity between nodes in different layers. Whereas we discuss latent patterns in the section Finding hidden interactions between two or more layers: Mediation, suppression, and other layer-interaction mechanisms below, here we focus on explicit interactions arising from connectivity patterns. Transitioning between multiple layers gives rise to multiple ways of defining connectedness in multilayer networks, see Fig. 2 (right). In single-layer networks, two nodes are connected if there exists a path between them (Newman, 2018). Analogously, several works on multilayer and multiplex networks defined connectedness as depending on the existence of a multilayer path between any two nodes (Battiston et al., 2014; Boccaletti et al., 2014; De Domenico et al., 2013). The largest connected component can then be defined as the largest set of nodes connected by at least one multilayer path (Battiston et al., 2020; Bianconi, 2018; Stella, 2018; Stella et al., 2018). This definition can be modified in the presence of explicitly defined inter-layer links (Bianconi, 2018). Importantly, the presence of several layers can give rise to additional definitions of connectivity that differ from their single-layer counterparts (Newman, 2018), thus giving rise to phenomena unobserved in single-layer networks, such as the identification of language kernels (Aitchison, 2012; Cancho & Solé, 2001).

The above multilayer definition of connectedness (Bianconi, 2018) exploits links present in either one layer or others. This combinatorial “OR” approach is different from requiring the presence of connected paths in all layers. Other works (Baxter et al., 2021; Baxter et al., 2014; Bianconi, 2018) considered *viability* as a definition of connectedness based on an “AND” logic: In a multilayer network, two nodes are viably connected if there exist intra-layer paths connecting those two nodes on every single layer. Intralayer paths are confined to a single path, for example, paths using links of only one color. A largest viable component (LVC) is the largest set of viably connected nodes (Baxter et al., 2021; Stella, 2020).

As highlighted in Fig. 2, the requirement of intra-layer connectedness across all layers (i.e., viability) is considerably more restrictive than the above definition of connectedness. This distinction naturally leads to the question of whether connected components and viable components might differ in their structure when modelling cognitive aspects of the mental lexicon (Stella et al., 2018), for example, contain different sets of concepts. Note that in single-layer networks, the largest viable cluster and the largest connected component (LCC) would be the same (Baxter et al., 2014). However, the LVC and the LCC would differ on multilayer networks made of different layers, potentially giving rise to phenomena unexpected in single-layer cognitive networks (Siew et al., 2019).

### The LVC corresponds to a spurt in language learning

The first quantitative evidence characterizing the cognitive relevance of LVCs was found by Stella et al. (2018). The authors identified an LVC of 1,000 words in a representation of the mental lexicon with an LCC of 8,000 English words, connected across four semantic/syntactic/phonological layers, namely free associations and phonological similarities from the MRC psycholinguistic database (Coltheart, 1981) and hypernyms and synonyms from WordNet (Miller, 1995).

All these layers were considered as binary and unweighted, i.e., two words were linked: (i) on the free-association layer if they were reminded of each other in a free association task (see also De Deyne et al., 2013); (ii) on the phonological layer if they differed by the addition/substitution/deletion of one phoneme (see also Vitevitch, 2008); (iii) on the synonym layer if they shared meaning in any given context (see also Lacasa et al., 2021); and (iv) on the syntactic layer if they were a more general instance (hypernym) or a more specific case (hyponym) of each other (see also Aitchison, 2012). By wrangling and combining such data from lexical repositories (e.g., synonyms, hypernyms/hyponyms, and phonological similarities) and from behavioral experiments (e.g., free associations), Stella et al. (2018) managed to build a multiplex lexical network representing binary unweighted associations between 8,000 English words. Weights were not considered (e.g., in terms of synonymity strength; Coltheart, 1981) because of technical issues in wrangling together, within the same network structure, wildly different sets of weights, and for avoiding the problem of choosing suitable heuristics for distinguishing between weak weights and absent interactions. However, Stella et al. (2018) conducted a preliminary analysis of such binarized multiplex network structure and – under the information theoretical framework of structural reducibility (De Domenico et al., 2015) – found that those four binary layers encoded different structural patterns between the same set of words: Each layer represented a different organization of conceptual associations in the mental lexicon. This motivated further analyses.

Notice that the 8,000 words in the multiplex lexical network included nouns, verbs, and adjectives already investigated in past psycholinguistic mega-studies by Brysbaert and colleagues, namely on age-of-acquistion (AoA) (Kuperman et al., 2012) and on concreteness (Brysbaert et al., 2014). Stella et al. (2018) used the psycholinguistic estimate of the age-of-acquisition of a given word to simulate how the network representation of the mental lexicon grew over time, i.e., by adding to the network one word after another, starting from those with lower AoA and proceeding in ascending order of AoA. The authors thus managed to simulate the growth of the mental lexicon and its LVC over time, according to the ordering of word acquisition indicated by AoA norms. Such ordering imitated the order in which most native English speakers acquire concepts over time, i.e., a normative acquisition as discussed in Steyvers and Tenenbaum (2005). Whereas the LCC grew smoothly over time, the LVC appeared with a sudden, discontinuous phase transition (also called explosive; Baxter et al., 2021) around age 7–8 years old, a critical age for the development of reading and reasoning skills in typically developing children (Frith, 2017; Piaget, 1964).

The LVC was also found to be rich in shorter and higher frequency/polysemy/concreteness words (compared to the LCC). When partitioning words as inside/outside of the LVC, each multiplex layer exhibited a core-periphery structure (Newman, 2018), with the LVC representing a network core, i.e., a set of tightly linked high-degree nodes connecting more peripheral low-degree nodes. Furthermore, when removing words in the LVC from the multiplex lexical network, the average network distance between words increased considerably more than removing words outside of the LVC but with a matched degree. Since distance in cognitive networks refers to conceptual similarities (Fatima et al., 2021; Siew et al., 2019), and shorter distance corresponds to quicker conceptual processing (cf. Kenett et al., 2017; Kumar et al., 2020; Vitevitch, 2019), this pattern indicates a beneficial role played by nodes in the LVC in providing shortcuts of conceptual associations between other concepts. Noticeably no LVC was found when the phonological layer was excluded from the analysis, suggesting that phonological associations are key to identifying the LVC. Given all of these characteristics of the LVC, Stella and colleagues suggested that the LVC was a language kernel in the mental lexicon (Cancho & Solé, 2001). That is, the LVC is a sample of highly frequent yet simple words that are prominent (e.g., well connected) in language and whose availability enables communicative advantages (Aitchison, 2012; Cancho & Solé, 2001; Gruenenfelder & Pisoni, 2009).

Interestingly, past approaches based on single-layer networks but on different datasets converged to results similar to the ones provided by multiplex lexical networks. Vincent-Lamarre and colleagues (Vincent-Lamarre et al., 2017; Vincent‐Lamarre et al., 2016) built a network of directed syntactic dependencies between words and others used to defined the latter across different English dictionaries. The authors identified language kernels as strong full connected components, i.e., sets of nodes that are mutually connected by directed sequences of connections (Newman, 2018). Despite these works (Vincent-Lamarre et al., 2017; Vincent‐Lamarre et al., 2016) using only connections induced by syntactic specifications – for example, in the sentence “love is weakness,” “weakness” is used to specify the concept of “love” – they found language kernels with the same order of magnitude of words of the kernels identified with multiplex lexical networks (Stella et al., 2018), which rather combine or overlap several relationships like the semantic, phonological, and syntactic ones defined above. Both approaches confirmed that words in the language kernel are learned earlier, are more concrete, and have higher frequency than others in the rest of the dataset/dictionary/network. The main advantage of multiplex lexical network is the fact that they do not require specific dictionary-like syntactic definitions (e.g., “water is a transparent liquid at room temperature”) and thus better appeal to the complex, intricate structure of the mental lexicon (Aitchison, 2012).

### The LVC identifies changes in mental navigation related to variation in creativity levels

A critical cognitive mechanism related to individual differences in creative thinking is associative thinking, which allows a creative person to search more broadly through their memory, leading to the combination of remote ideas into original and appropriate ideas (Beaty & Kenett, 2023). Thus, a complementary question is: Can modelling how a person searches their memory be used to predict how creative they are? This is indeed possible via cognitive multiplex network modelling.

This exact question was examined by Stella and Kenett (2019), who reanalyze data collected by Kenett et al. (2016). In the original work by Kenett et al. (2016), a large sample of participants underwent an animal-category semantic fluency task, tasks assessing their fluid intelligence, and a self-report questionnaire assessing creative achievements (Carson et al., 2005). The authors then divided the sample into four groups – low/high creativity and low/high intelligence – and estimated their group based semantic memory networks.

To maximize group differences, Kenett and Stella (2019) only analyzed the two extreme groups from Kenett et al. (2016) – low creativity/low intelligence and high creativity/high intelligence. Stella and Kenett (2019) analyzed partipants’ performance in a semantic fluency task, as an operationalization of a mental navigation task that operates over memory when searching internally (Abbott et al., 2015; Ovando-Tellez et al., 2022; Todd & Hills, 2020). In this task, participants are required to generate as many category members as possible, in a given amount of time. Computational methods allow examining how people search through their memory (Benigni et al., 2021; Hills et al., 2012; Todd & Hills, 2020), tracing the paths they traverse over representations of their mental lexicon (Hills & Kenett, 2022). Often, this task is based on the animal category (Ardila et al., 2006; Hills et al., 2012), i.e., name as many animals as possible in 2 min. Specifically, Stella and Kenett (2019) only focused on the creativity scores of participants in both groups, investigating how the mental navigation of these participants can be utilized to classify them into their creativity group (lower or higher creativity).

**Fig. 3:**
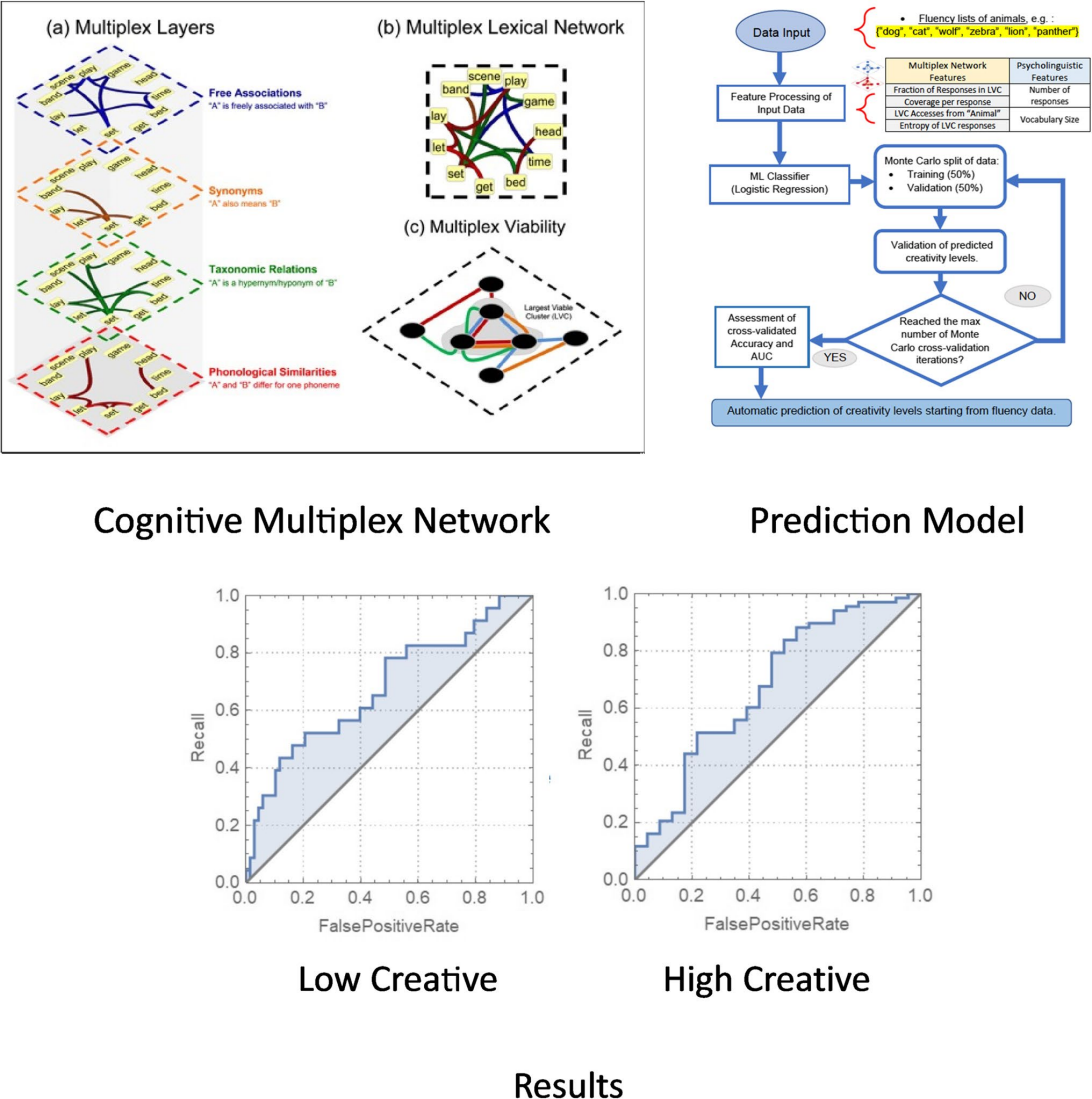
Illustration of the process followed by Stella and Kenett (2019). First, the authors constructed a cognitive multiplex network (**top left**). Next, the authors quantitatively examined participants’ semantic fluency responses as a mental navigation process over the cognitive multiplex network (**top right**). Participants’ measures of how they explore the cognitive multiplex network when searching for animal names was then used to build a machine learning model to classify participants in low or high creative individuals. This predictive model was accurate up to 75% of the time in classifying participants into their correct group of low- and high-creative individuals (**bottom**)

To classify participants as lower- or higher-creative individuals, the authors used a multiplex network to represent lexical memory over the same four layers used in Stella (2018), but extended it to 16,000 English words. This multiplex network exhibited an LVC with over 1,100 words (see Fig. 3). Stella and Kenett examined computationally the way people exploited their memory, and classified participants into low- and high-creative individuals, based on the way they “walked” on the multiplex network, through the LVC. In other words, the authors analyzed participants’ measures of navigating over the cognitive multiplex network focusing on the LVC.

The authors found that the lower- and higher-creative individuals differed in several cognitive multiplex measures, largely focusing on how they rely on their performance on the LVC, and on the number of responses they are able to generate. Individuals with lower creativity accessed the LVC considerably more than those with higher creativity, suggesting a beneficial role for the LVC to support recall in people unable to employ other cognitive strategies to achieve higher levels of creativity. Such distinctive patterns were measured through network access, distance, and entropy, and became a set of features in an artificial intelligence (AI) model that was trained to categorize high/low creativity levels from network measures of LVC access. In a leave-one-out cross-validation, the AI achieved an accuracy of 65 ± 1% (compared to a baseline of 50% for random guessing), indicating that multiplex viability may influence conceptual recall and high-level cognitive strategies related to creativity (Hills & Kenett, 2022; Kenett et al., 2018).

Overall, the work of Stella and Kenett (2019) is significant in two ways. First, it demonstrates how a viable component in a multiplex network can be used to examine complex cognitive processes, such as mental navigation operationalized via a semantic fluency task. Second, the LVC can be used to extract features and construct machine-learning predictive models, correctly classifying individuals’ creativity levels based on fluency data, for example, mentioning synonyms of “hot” or animals from the animal category within 2 min. Such evidence opens up the door to additional, future studies in representing complex cognitive processes and their interactions with personality traits (Samuel et al., 2023; Stella & Kenett, 2019).

### The LVC supports correct picture naming in people with aphasia

Another example illustrating an advantage for words in the LVC comes from picture naming in people with aphasia. In a picture-naming task individuals are visually presented with a line drawing or photograph and asked to name the object that is depicted, a task formalized in the Philadelphia Naming Test (PNT), a 175-item picture-naming test developed in the Language and Aphasia Lab of MRRI (Roach et al., 1996). Aphasia describes a spectrum of language disorders, impacting word processing, understanding, and production (Dell et al., 1997). Castro et al. (Castro & Stella, 2019; Castro et al., 2020) investigated picture naming through a multiplex lexical network with 8,000 words linked by free associations, hypernyms/hyponyms, phonological similarities, and synonyms. The authors found that multiplex distance was important for predicting not only the rate of correct picture naming (Castro & Stella, 2019), but also the types of mistakes made by people with aphasia (Castro et al., 2020). Higher predictive power in the first (regression) and second (multinomial regression) tasks was achieved when multilayer distances were used, rather than considering network layers in isolation.

Building on those findings, Stella (2020) discovered that items in the LVC were named correctly with frequency rates at least 30% higher than for items found outside of the LVC. Further, through network attacks accounting for frequency, degree, and word length effects, the author found that the probability of correct production in PNT could efficiently identify words within the LVC (compared to random guessing; Stella, 2020), fully dismantling the LVC by removing only one-third of its nodes. Together, these results suggest that words in the LVC might benefit from enhanced lexical retrieval mechanisms that in clinical populations lead to more accurate production of words in the LVC (as measured by the PNT; Stella, 2020), and in healthy populations supports recall in individuals with lower creativity levels (Stella & Kenett, 2019) and lower openness (Samuel et al., 2023).

To sum up, in multilayer representations of the mental lexicon, viability can identify a language kernel that has interesting features for cognitive processing. This kernel emerges from the interactive nature of semantic and phonological associations (Levy et al., 2021; Stella et al., 2018), and facilitates cognitive processing in both healthy and clinical populations. Importantly, it is not possible to identify such a kernel in single-layer networks that model only part of the mental lexicon (Stella, 2019; Stella et al., 2017; Stella & Kenett, 2019), highlighting the importance of using the multilayered network approach to shed light on cognitive representation and processing. Future research should further investigate clusters like the LVC and identify new ones, potentially arising from other types of relationships among words or by including other pieces of lexical information in the network (Citraro et al., 2023). The clusters that are discovered in multilayer networks could lead to novel insights and provide quantitative ways to examine cognitive processing (Vitevitch & Mullin, 2021), creativity (Beaty et al., 2023; Benedek et al., 2023), cognitive functions in altered states of consciousness (Rastelli et al., 2022), and language acquisition (Citraro et al., 2023), among other processes relevant to cognitive network science (Castro & Siew, 2020; Siew et al., 2019).

#### Community detection in multilayer networks highlights shortcuts between semantic themes

A community is a group of nodes more closely or tightly connected to each other than to nodes belonging to other groups (Blondel et al., 2008). Community detection is the task of decomposing a network into well-connected and well-separated groups of nodes, and it is one of the most challenging problems in complex network analysis (Fortunato, 2010), in part because of the different topological criteria adopted to define a “community” (Newman, 2018). Community detection algorithms, used to identify communities in a network, can be classified according to the way they approach the community-detection task (Blondel et al., 2008; Girvan & Newman, 2002; Palla et al., 2005). The most common algorithmic approaches used to partition single-layer networks fall into two classes, adopting either (i) the well-known modularity-based optimization scheme (Blondel et al., 2008) or (ii) the concept of k-cliques – subgraphs where all nodes are adjacent to each other and have degree *k* – to extract sets of overlapping communities (Palla et al., 2005). Both community detection approaches have found clusters of words sharing similar linguistic traits in single-layer networks, like shared sequences of phonemes in phonological networks (Siew, 2013), or concepts falling in the same semantic field (found in a free association network; Palla et al., 2005) and in a syntactic network (Gerow & Evans, 2014).

The task of community detection is more complicated in multilayer networks, because the community detection algorithm must consider the different types of relationships occurring in different layers at the same time (Magnani et al., 2021). Multilayer community detection algorithms are classified according to the strategy chosen to handle the presence of multiple layers:

- Flattening methods reduce all layers into a single one, making the structure suitable for classic community detection (Berlingerio et al., 2011). This approach was used by Vuki´c and colleagues, who identified different semantic fields of “database” via community detection in a multilayer network with factual, conceptual, procedural, and metacognitive connections between concepts (Vukić et al., 2020).
- Layer-by-layer methods process layers independently before merging the final list of communities through consensus, (Magnani et al., 2021).
- Multilayer methods act directly on the multilayer structure, finding communities by transitioning across the layers. In this class of methods, we find extensions of the modularity-based approaches in single-layer networks to multilayer networks. Examples of this approach include GLouvain (Mucha et al., 2010) or the multilayer extension (Edler et al., 2017) of the Infomap algorithm (Rosvall & Bergstrom, 2008).

Choosing the most suitable community-detection method for a multiplex or multilayer lexical network is a challenge to cutting-edge research in cognitive networks. Only a few works have indirectly addressed this problem so far. Kov´acs et al. (2021) analyzed the multilayer network structure of semantic, syntactic, and phonological associations of several languages, including English and Hungarian. Using a modularity maximization approach, they found that larger communities tended to reflect mostly semantic associations, whereas smaller communities encoded encyclopedic knowledge (Kovács et al., 2021).

Interestingly, the task of grouping nodes in a multilayered network is tied to the task of detecting communities in attributed networks, where nodes also possess categorical information or features (Chunaev, 2020). Notice that attributed networks are graphs, i.e., collections of nodes linked together, where either nodes or links can be labelled with categorical or continuous data, for example, nodes representing words might be labelled as “positive,” “negative,” or “neutral” according to their valence (Scott et al., 2019; Stella, 2022) or be attributed their age of acquisition, a numerical average score in years as obtained from a psychological mega-study (Citraro & Rossetti, 2020; Steyvers & Tenenbaum, 2005).

In this vein, Citraro and Rossetti (2020) recently tackled community detection in multilayer lexical networks by introducing the Extending to Vertex Attributes Louvain or EVA method. Their approach extends the modularity-based optimization function (Blondel et al., 2008) to a multi-objective criterion requiring communities to be as homogeneous as possible to the features carried by the nodes and across different network layers. The idea of such a requirement comes from the well-documented tendency for conceptual associations to be present between concepts sharing similar features (Aitchison, 2012; Kenett et al., 2017; Kennington & Schlangen, 2015; Kumar et al., 2020). Requiring communities to be homogenous, or to contain as many as possible words with the same feature, allows one to obtain communities of words/concepts that are more consistent with the relevant literature about the mental lexicon. The authors tested the multiplex mental lexicon built in (Stella et al., 2018) and enriched it with lexical features such as word length, valence, arousal, dominance, semantic size of denoted words, and gender association (from the Glasgow dataset; Scott et al., 2019).

### Multiplexity highlights shortcuts between concept communities in a phonological/semantic multiplex model

**Fig. 4:**
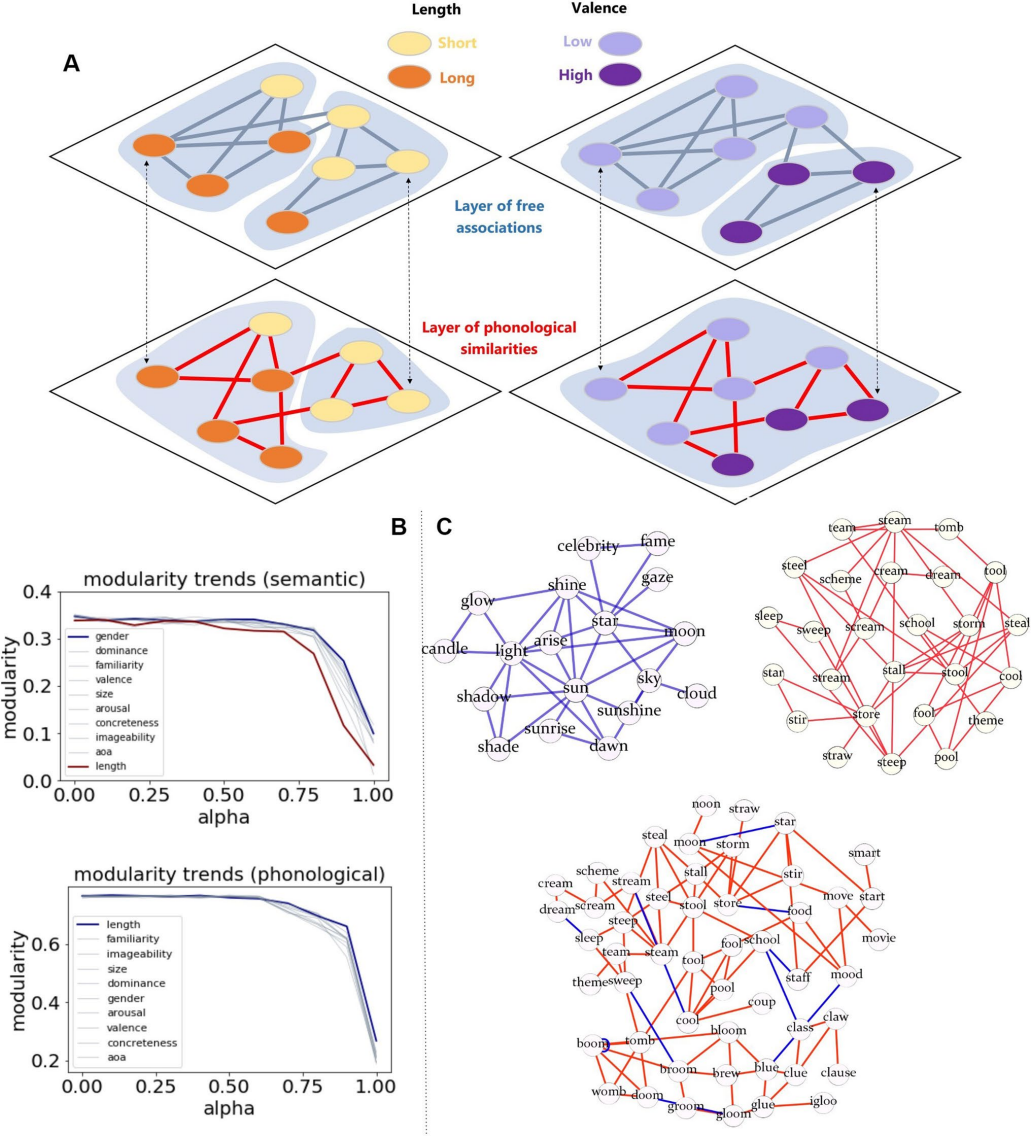
(**A**) Attribute-aware community detection in a toy multilayer lexical network: a toy partition for word length (**left**) and valence (**right**). Results from Citraro & Rossetti (2020). (**B and C**) Different attributes correlate with different layers (**B**); a toy example of matched communities on the semantic (blue links), phonological (red links), and multiplex (mixed links) lexical structure for the arousal property (**C**)

Figure 4(A) sums up with a toy example what community detection algorithms that are also sensitive to the features attributed to each node can reveal in multilayer lexical networks. Analyzing the aggregated multiplex structure obscures the variation in feature homogeneity across layers. For instance, when requiring communities to feature words sharing as much as possible the same word length, modularity shows a very fast decrease in the semantic layers, but remains stable in the phonological layer (Fig. 4B). This means that the semantic layer is unable to provide communities where most words have the same length, whereas the structure of the phonological layer can provide such communities. This dichotomy essentially reflects the absence of a clear cognitive constraint influencing the layout of conceptual associations in the semantic layer based on length. Instead, in the phonological layer, where shorter words tend to share connections, the edit-distance rule – grounded in several research studies (Chan & Vitevitch, 2009; Siew, 2013; Vitevitch & Mullin, 2021) – creates several communities where words share the same length, and the algorithm can thus identify such solutions even at higher resolution powers.

Two main results emerged from the feature-rich multiplex mental lexicon (with 4,000 words and two layers, i.e., free associations and phonological similarities) analyzed by Citraro and Rossetti (2020):

1. Communities extracted by EVA reflect thematic contexts: Concepts can fall within different contexts according to the psycholinguistic features used to perform community detection, even keeping the network fixed. In other words, one network can give rise to many sets of communities according to the feature selected for EVA. For instance, “star” belonged to a community of words relative to astrophysics when semantic size was used as a feature for community detection on the semantic layer. On the same layer, “star” belonged to a community of words relative to “shining” when arousal was used instead (Fig. 4C). Such thematic coherence and context swapping were not observed in the phonological layer. These findings indicate an important interplay between semantic features (e.g., “being astrophysics objects,” “shining”) and semantic layers in multiplex networks, which should be treated differently from phonological similarities. While it is intuitively expected for semantic features to influence thematic coherence more prominently on semantic rather than phonological networks (Aitchison, 2012), these quantitative models open the way to mapping and exploring the interplay between psycholinguistic norms and network structure (Citraro et al., 2023).
2. As illustrated within Fig. 4C, semantic links provide shortcuts between different clusters of phonologically similar words that would otherwise be at a greater network distance. This happens because the homogeneity of the same feature (e.g., arousal) over communities spanning different layers changes significantly across layers. Although less thematically cohesive, the multiplex network structure provides shortcuts that make single-layer communities overlap with each other. Recent investigations indicated that semantic and phonological connections are systematically better than random links in decreasing the average network distance between words (Levy et al., 2021). Hence the observed overlap in communities from different layers might be due to a potential cognitive benefit, worthy of further research.

#### Finding hidden interactions between two or more layers: Mediation, suppression, and other layer-interaction mechanisms

It is only recently that current research in multilayer networks has highlighted quantitative frameworks for capturing mediation and suppression between network layers (Lacasa et al., 2021). In quantitative psychology, mediation represents the consideration of how a given variable D affects the causal relationship between two other variables A and B (MacKinnon et al., 2007). In the presence of causal chains, *A* → *D* → *B*, D acts as a mediator variable. Accounting for the mediator variable D might reduce or weaken the direct relationship, when present, between A and B, since the mediator might explain a part of the influence exerted by A over B. In other cases, the addition of a third variable does not reduce it but rather restores or amplifies the influence exerted by one variable over a second, dependent, one. In these cases, the third variable provides a suppression effect (MacKinnon et al., 2000).

The key research question in capturing mediation/suppression in multilayer networks is: Does the topology of a given layer depend on the structure of another layer but through the mediation/suppression of a third unseen network layer? This research question is evidently different from capturing directly the similarity between the organization/topology of any two layers, a task that can be performed by using a wide variety of spectral and information-theoretic metrics (De Domenico & Biamonte, 2016; De Domenico et al., 2015; Hartle et al., 2020; Mheich et al., 2020; Santoro & Nicosia, 2020). The recent findings of Lacasa et al. (2021) relied on capturing mediation/suppression over the individual links, potentially replicated or not, across any two network layers. Their approach has not been applied to cognitive networks yet. Nonetheless, it represents an interesting direction for future research in psychological representations of the mental lexicon.

To better understand how such machinery would work in multiplex cognitive networks, let us consider the example of a two-layer multiplex network with a free-association layer and a phonological layer, analogous to the ones discussed in Levy et al. (2021) or Citraro and Rossetti (2020). These different layers might display a small level of correlation since words sounding similar to each other tend also to be recalled together in free-association tasks (as measured with link overlap in Stella et al., 2017). However, this similarity might be due to causal relationships: Sound similarity might facilitate or inhibit a memory recall pattern (Vitevitch & Mullin, 2021). Alternatively, this influence might not be directly evident. Analogous to latent variables in psychometrics (Golino et al., 2022), there might be a hidden network layer of conceptual associations that either confounds or mediates the relationship between phonological similarities and free associations or other pairs of layers in the mental lexicon.

The recent study by Lacasa et al. (2021) introduces a framework to quantify mediation and suppression between networks. The framework works at the level of edges ${e}_{ij}^{C}$ between words *i* and *j* on layer *l*. Layers are considered as variables, so that a layer *l* = *A* might exert a causal influence over another layer *l* = *B* through the mediation or suppression of a third layer *l* = *C*. In the case of mediation, the presence of a link ${e}_{ij}^{C}$ in the hidden layer would also determine the presence of the same link in layers A and B. In the case of suppression, the presence of a link ${e}_{ij}^{C}$ in the hidden layer would determine the presence of the same link only in layer A (but not in B) or only in layer B (but not in A). Counting edges can give rise to an information-theoretic framework inferring the presence of a mediation/suppression mechanism, purely based on multilayer network structure and link correlations (for the mathematics, we refer the reader to Lacasa et al., 2021).

The applications of such network-based mediation/suppression framework have to date involved social networks (online, professional, personal interactions) and mesoscale connectomes in the complete *C. elegans* nervous system (Lacasa et al., 2021). In lexical networks this approach could address questions such as: “Are semantic associations more likely to occur if there is semantic overlap but not phonological similarity?” If we consider factual and metacognitive layers in a knowledge network, this approach could be combined with the multiplex representation introduced by Vukić et al. to identify mediation and suppression mechanisms in processing domain knowledge (Vukić et al., 2020).

Neither of the above examples could be investigated with single-layer networks, highlighting the importance of using multilayer networks and mediation and suppression techniques to detect latent relationships in data due to layer-interaction mechanisms.

A concrete example of the relevance of layer-interaction mechanisms in exploring cognition comes from recent multilayer investigations of the issue of lexical access (Levy et al., 2021). Please note that we are considering lexical access in the context of comprehension and recognition processes where word recognition, unlocking or accessing the meaning of a word requires a flow of information from phonology to semantics (Aitchison, 2012; Vitevitch & Mullin, 2021). Classic linguistic theories assume that in order to comprehend or produce meaningful linguistic output, one needs to access and retrieve information from their mental lexicon, a process known as *lexical access* (Aitchison, 2012). Lexical access involves multiple processes of representation, in particular, a semantic word-meaning process and a phonological wordform mapping process, that allow access and retrieval from the mental lexicon (Dell et al., 2014). However, whether the relation between these two processes is serial, parallel, or interactive is still debated (Dell et al., 2014; Nadeau, 2012). The modular account argues for a detailed process between two discrete modular processes of lexical access. According to this account, during lexical access of a linguistic input, semantic processing takes place only after phonological processing is completed. The cascading account argues for a more relaxed modular account. According to this model, semantic processing can initiate before phonological processing is complete. Finally, the interactive model theorizes that lexical access involves an interactive spread of information across a phonological layer and a semantic layer that can influence each other (Dell et al., 2014; Dell et al., 1997). This model argues that both layers are structured as a network, and that information spreads across these two networks, related to the organization of concepts across both layers and to the strength of links that connect them. However, the specific model of lexical access is still open for debate.

### Layer-interaction mechanisms in a phonological/semantic multiplex network

**Fig. 5:**
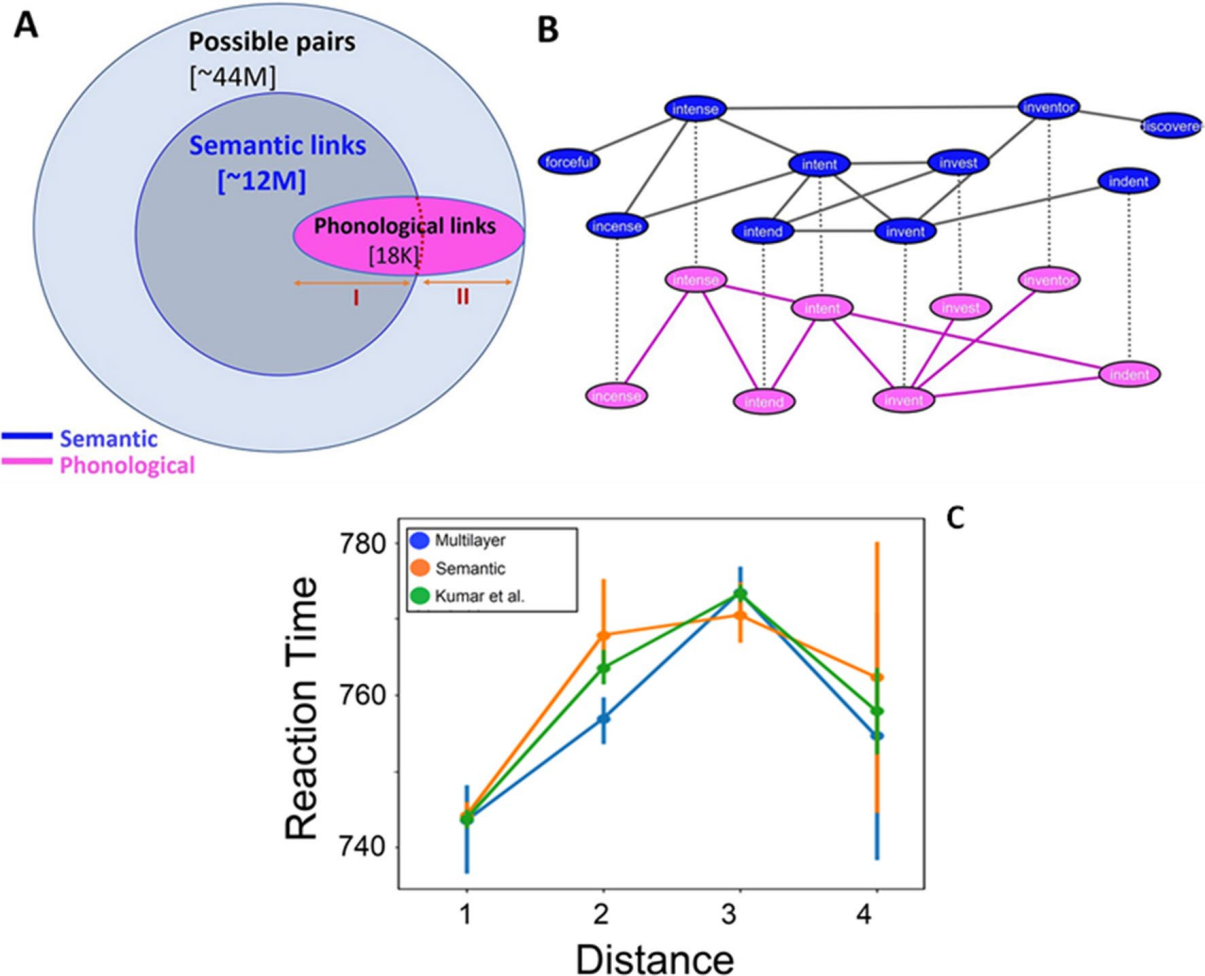
**Top:** Multiplex network of semantic and phonological layers constructed by Levy et al. (2021). (**A**) Illustration of the overlap across the (weighted by relative frequency with which a given association was produced by participants) semantic layer and the (unweighted) phonological layer. (**B**) Illustration of the multiplex network with nodes and links across both semantic and phonological layers.** Bottom:** The multilayer network is more quickly accessible than the semantic network (**C**). Reaction times (RTs) for relatedness judgments in the Kumar et al. (2020) network (Green), Levy et al. (2021) semantic network (Orange), and the Levy et al. (2021) multilayer network (Blue). Error bars for the Levy et al. (2021) networks represent standard deviations of the average RT. The error bar of the Kumar et al. (2020) network represents the standard deviation of averages of subsets of RTs

In a recent study, Levy et al. (2021) applied a cognitive multilayer network analysis to directly analyze and quantify the relation between phonological and semantic networks, motivated by the interactive model of language processing (Dell et al., 2014). To do so, the authors constructed a large-scale multilayer network comprised of empirical phonological and semantic layers (Fig. 5), for a large-scale network of about 9,000 words [18]. The authors then conducted the following analyses: First, they examined the similarity between the two layers by measuring their link overlap. Next, they measured the effect of adding non-overlapping links from one layer to the other. Finally, Levy et al. examined the potential benefit of combining both layers as a multilayer network on lexical access, by measuring the networks’ average distances of the single layers versus the multiplex (Levy et al., 2021).

Indeed, in their approach, Levy et al. (2021) considered “interactive” the emergence of shortcuts in the whole multiplex network benefiting from both phonological and semantic links. They highlighted such interactiveness in two ways, without ever considering mediation/suppression effects (which we covered in the section Finding hidden interactions between two or more layers: Mediation, suppression, and other layer-interaction mechanisms instead). In a given multiplex layer, it is expected for the addition of non-overlapping links to decrease the overall distances between concepts: New links can create novel shortcuts and these can be beneficial for mental search processes, as we discussed already above. A key question becomes whether this reduction happens at faster or slower rates depending on whether these links are added according to a certain strategy. Through simulations, Levy et al. (2021) showed that adding links at random shortened the distance between words at much slower rates/smaller magnitudes compared to adding those same links present in one layer to the other. Importantly, adding links from word-pairs that had short paths in one layer largely reduced distances of word pairs that had short distances in the other layer. Thus, adding links from one layer to the other had a specific, non-random effect.

Furthermore, Levy et al. empirically demonstrated the significance of their notion of interactiveness of the multilayer architecture. They did so by reanalyzing response time (RT) data collected by Kumar et al. (2020). Kumar et al. (2020) computed a single-layer semantic network in English based on the University of South Florida Free Association Norms, comprising 5,000 normed words (Nelson et al., 2004). Kumar et al. (2020) demonstrated how semantic distance based on path length in their network related to participants’ reaction time (RT) while judging whether word-pairs are related to each other. In doing so, Kumar et al. replicated and extended similar findings previously found in Kenett et al. (2017). To do so, Kumar et al. had participants make relatedness judgments on pairs of cue words that varied in the semantic distance between the words based on the path length in their semantic network for those two words (Kumar et al., 2020). Levy et al. (2021) conducted the following analysis:

First, they identified links from the Kumar et al. semantic network that corresponded to links in their semantic layer. Next, they examined how semantic and multilayer path lengths related to RT in the corresponding links from the Kumar and colleagues’ study. Critically, Levy et al. found that short multilayer path length related to shorter RT compared to the same path length (or two steps) in the semantic network of Kumar et al. as well as the semantic layer of Levy and colleagues (Fig. 5). Thus, Levy and colleagues empirically demonstrated the cognitive advantage of conceptual paths between concepts in the mental lexicon based on “interactive” relations of phonological and semantic information.

Thus the authors argue that the interaction between these two layers might be crucial for allowing more efficient lexical access, by reducing path distances between nodes in a cognitive multilayer network (Levy et al., 2021). For example, in the phonological layer, the cue words *intend* and *invest* had a path distance of three (intend → intent → invent → invest). However, in the semantic layer, these cue words *intend* and *invest* are directly connected, see Fig. 5B. Thus, in a multilayer phonological-semantic network, the distance between intend and invest is much shorter than in a phonological only network, enhancing the lexicon’s efficiency in lexical access even in potential impairments (Castro & Stella, 2019; Castro et al., 2020).

Overall, the multilayer approach by Levy et al. demonstrates the strength of applying a cognitive multilayer network analysis to examine classic cognitive theories, such as on the nature of lexical access (Levy et al., 2021). It also demonstrates the feasibility of combining computational modelling with empirical research to advance cognitive research.

## Discussion, limitations, and future directions

Recent work using multilayered networks in cognitive science has revealed key insights that would not have been observed using single-layer networks. This work has examined cognitive processing in healthy (Levy et al., 2021; Samuel et al., 2023; Stella et al., 2017; Stella et al., 2018; Stella & Kenett, 2019) and clinical populations (Castro & Stella, 2019; Castro et al., 2020), revealed clusters and communities reflecting different contexts and meanings of individual concepts (Citraro & Rossetti, 2020; Citraro et al., 2023; Dell et al., 1997; Kovács et al., 2021), and discovered latent mediation/suppression interactions between different aspects of knowledge (Dell et al., 1997; Marinazzo et al., 2022). These findings illustrate the potential for multilayer networks to advance the cognitive sciences using a quantitative, interpretable, and human-centric framework. Multilayer networks give structure to the representations found in interactive layers of the mental lexicon (quantitative; De Domenico, 2022). This structure can be interpreted using various network measures, such as network distance and concept relatedness (interpretability, e.g., Kenett et al., 2017; Kumar et al., 2020), and may account for the complexity of the human mind (human-centric; Stella, 2019).

Although the use of multilayer networks has much potential, this approach also has some limitations, which naturally lead to crucial directions for future research. For example, in most cases (including the examples presented here) links are defined between pairs of nodes. In some cases, it may be more useful to create a set of nodes instead of simply pairs of nodes to form a hypergraph. For example, several actors feature in scenes in movies without just co-occurring with each other, or more than two words in a sentence might modify its meaning. Hypergraphs are being increasingly studied and applied (Battiston et al., 2020) to account for simultaneous interactions between more than two entities at once. From a cognitive perspective, hypergraphs could be structured across multilayer/multiplex structures either by building links through information-theoretic measures (Marinazzo et al., 2022) or by considering other interaction patterns between concepts (e.g., phonological similarities between words sharing the same skeleton of vowels and consonants; Gruenenfelder & Pisoni, 2009). In either case, future research using the hypergraph approach could potentially reveal higher-order behaviors that are not observable with pairwise relationships, perhaps identifying communities of concepts reflecting specific semantic fields (Gerow & Evans, 2014) or contexts of usage (Citraro et al., 2023).

Another limitation comes from the fact that by merging different weighted layers, an experimenter might need to select different strategies for distinguishing between present, weak, and absent relationships between words in the data. These strategies might have to deal with different weight distributions across each layer and would require some motivation in terms of cognitive modelling. These aspects represent important modelling challenges, which might be addressed by taking inspiration from renormalization theories in regression models (e.g. lasso regression or ridge regression (Fu, 1998) and lead to future frameworks accounting for weighted similarities or connections between words (an interesting early stage approach being the one developed in Citraro et al. (2023), but only for categorical variables).

Multilayer networks contain several network layers, and thus correspond to an increased chance of mistakes in assessing whether two concepts should be connected or not, such as whether two concepts are syntactically related in speech (Morgan et al., 2021; Parola et al., 2023) or in text (Semeraro et al., 2022). Mechanisms for link prediction or noise correction should thus be applied to next-generation multilayer models of the mental lexicon. Bayesian inference can identify errors in a given network layer even in the presence of unknown and heterogenous uncertainty (Peixoto, 2018). The formalism proposed by Peixoto checks for the presence of connections between different clusters of nodes, using structured generative network models to infer the presence/faulty presence of individual links even without direct error estimates. This approach could filter layers of free associations (De Deyne et al., 2013) or other types of semantic/syntactic datasets (Quispe et al., 2021), lessening the impact of noise over the multilayer structure.

Single-layer and multilayer networks are useful constructs to make sense of complex and multivariate systems (Newman, 2018), but they remain modelling proxies. The mental lexicon might not look like a network in some specific instances, as recently discussed by Hills and Kenett (2022). Alternative modelling approaches should thus be pursued in parallel with multilayer networks, leading to next-generation studies where multiple models are compared or used together. Word embeddings (Kennington & Schlangen, 2015; Kumar, 2021; Kumar et al., 2022; Litovsky et al., 2022) represent promising alternative modelling approaches, giving more emphasis to the vector-like nature of features associated with individual concepts. Frameworks encompassing vectors and multilayer networks to model the mental lexicon, like the FERMULEX approach by Citraro and colleagues (Citraro et al., 2023), represents an interesting attempt to capture the complexity of mental representations.

Building network models that minimize redundant features while maximizing informativeness (e.g., prediction power about word norms; Scott et al., 2019) under potential uncertainty is becoming increasingly relevant in quantitative psychology, especially network psychometrics (Christensen et al., 2023; Golino et al., 2022).

A current limitation of cognitive multilayer networks is the selection of which layers to include and which to discard when building a representation of the mental lexicon (Citraro et al., 2023). Adding more network layers can provide more information about connectivity patterns between concepts but, at the same time, it can add unnecessary redundancy (Santoro & Nicosia, 2020). This should be circumvented by a data-informed selection of layers encoding conceptual associations being relevant for the cognitive phenomena being investigated. For instance, when investigating iconicity (Aitchison, 2012), one should consider both semantic and phonological associations for mapping word forms and meanings together. However, there are several ways to encode a given type of conceptual association, for example, phonological similarities might be captured in different ways according to different ways of achieving phonological transcriptions. This data issue naturally leads to hundreds, if not thousands, of possible network layers composing a cognitive multilayer network.

One could challenge the cognitive plausibility of such rigid distinctions – would humans encode hundreds of distinct and potentially minimally different layers in their mental representations of associative knowledge? Cognitive experiments (Dóczi, 2019) indicate that associative knowledge is resilient to noise and might thus not be encoded in strict ways, for example, “parser” and “parsing” would sound similar and appear as sharing some meaning to a human despite not being directly linked in a phonological network. This evidence suggests that network layers might differ in non-trivial ways, not because of the absence/presence of a few different connections between concepts, but rather because of some more general structural patterns. Fewer but maximally different network layers might be cognitively plausible, at least in some instances of knowledge processing (Hills & Kenett, 2022). These would be the outcome of a feature economy process, apt at communicating effectively with limited cognitive resources (Aitchison, 2012).

Cognitive multilayer networks might thus open the way to novel exciting future research aimed at assessing, quantifying, and understanding both universal similarities and individual differences in how humans can integrate multiple nuanced but analogous similarities along multidimensional cognitive representations of associative knowledge. However, how can future research quantify whether distinct layers in multilayer models are merely data-driven or rather constitute accurate cognitive representations? This limitation can be addressed in two ways. First, representations of the mental lexicon should include only the layers that are relevant for a specific task. For example, to model the process of reading the interplay between phonology and orthography requires both layers (Siew & Vitevitch, 2019). Second, once relevant layers have been selected, additional tools from information theory can quantify the amount of redundant information embedded in a given combination of layers. Structural reducibility analysis (De Domenico et al., 2015) and compressibility (Santoro & Nicosia, 2020) can identify the best combination of network layers maximizing information gain compared to a baseline model where all layers are aggregated together. Information gain could be implemented in different ways (Santoro & Nicosia, 2020). In multiplex networks, the maximization of the Von Neumann entropy was shown to successfully identify those layers providing the most information about node connectivity (De Domenico et al., 2015). The experimenter should thus check whether a preliminary representation of the mental lexicon could be further aggregated or compressed via entropy maximization, which would indicate the presence of redundant layers to be aggregated with each other to limit the number of layers to be considered. These approaches have unveiled that many social and technological multilayer networks exhibited moderate redundancy (De Domenico & Biamonte, 2016; De Domenico et al., 2015; Santoro & Nicosia, 2020) and could be further reduced/compressed in structure. This was not the case for all multiplex lexical networks reviewed here, which showed how semantic, syntactic, and phonological aspects of words captured very different, and thus irreducible, patterns of connectivity (Stella et al., 2017; Stella et al., 2018; Stella & Kenett, 2019). Future research should combine informed designs and information-theoretic tools to better select appropriate layers for and reduce redundancy in a given multilayer model of the mental lexicon.

Attention has to also be paid to the issue of model interpretability. In the research papers we reviewed here, networks give structure to psychological data, thus acting as a “modelling prism” that captures the structural organization of data, for example, the presence of long-distance paths between word pairs (Citraro & Rossetti, 2020; Levy et al., 2021) or the location of words inside or outside from a special connectivity cluster such as the large viable component (Samuel et al., 2023; Stella et al., 2018). Knowledge about these structural patterns provides more control to the experimenter using these features for machine learning. The data patterns highlighted and unveiled by network structure should be used as features in interpretable AI frameworks (Molnar, 2020), i.e., frameworks where the directionality and impact of features over AI performance can be assessed, tested, and identified – for example, as in SHAP scores. Recently Baker et al. (2023) used SHAP scores to identify how shorter distances on a free-association layer and longer distances on a phonological layer might help an AI model recognize semantic mistakes in picture-naming tasks. The authors related these directional patterns to a spreading activation framework (Collins & Loftus, 1975; Siew, 2019), with network distance capturing how related words are in exchanging activation signals within the mental lexicon structure. The authors interpreted their findings as activation spreading taking place on multiple levels but failing mostly in semantic aspects of the mental lexicon, and leading to naming a word semantically related to the target one, i.e., producing a semantic mistake (for more details, see Baker et al., 2023). This example underlines how the additional knowledge provided by networks as “modelling prisms” and the adoption of interpretable AI models, should both promote a next-generation adoption of interpretable, testable, reproducible, and quantitative frameworks for cognitive modelling. Notice that simply putting data without the control and additional knowledge provided by network structure would greatly reduce and impair model interpretability, since the experimenter would not have direct access to the features being spotted and captured by the AI (e.g., network distance capturing spreading activation aspects).

Importantly, networks are not only being used to understand the complexity of the human mind (Hills & Kenett, 2022; Stella, 2018; Vitevitch, 2008), but are also being employed to understand the complexity of the human brain (Aerts et al., 2016; Amico et al., 2021; Betzel & Bassett, 2017; Bullmore & Bassett, 2011). These single-layer networks of the brain may connect brain regions that are physically connected or brain regions that are active at the same time. An ambitious goal for future research is to use the multilayer network approach to connect the cognitive network layer to the brain network layer to finally bridge the intangible mind and the physical brain (Vitevitch & Mullin, 2021; Zaharchuk & Karuza, 2021). At present, it is not clear how many network layers would be needed to accomplish this, or what those intermediate network layers might represent. It is also not clear if the spread or diffusion of activation that is commonly used to model cognitive processing in cognitive network models (Litovsky et al., 2022; Vitevitch & Mullin, 2021) is an appropriate mechanism to model the processes that occur at other network layers. Connecting the mind and the brain using multilayer networks may seem like an elusive goal, but we need only look to the Internet for existence proof of a physical network (i.e., the fiberoptic cables that envelope the world) bridging to the intangible social networks that emerge on software platforms like Facebook, Twitter, etc. (whose information is transmitted across those fiberoptic cables). Future research bridging cognitive and brain networks within multilayer, feature-rich frameworks might contribute to building a quantitative understanding of how the brain stores conceptual representations of words, which represents an intriguing brain/mind puzzle (Poeppel & Idsardi, 2022).

## Additional materials for psychologists interested in learning more about multilayer networks

Multilayer cognitive networks remain within the realm of cognitive network science, a relatively novel topic for psychologists that is quickly growing within the community (Castro & Siew, 2020; Siew et al., 2019; Stella et al., 2022). For this reason, it is important to provide a concise methodological complement to the psychology and cognitive science papers reviewed above. This subsection includes a selection of relevant methodological papers, reviews, tutorials, and programming tools that can be adopted by readers interested in applying multilayer networks to their multidimensional psychological data.

The rapid growth of the field motivated the appearance of several relevant reviews about cognitive networks in psychology (Castro & Siew, 2020; Siew et al., 2019) and in cognitive data science (Hills & Kenett, 2022; Stella, 2022), which can all be interesting starting points for learning more about network measures and their psychological interpretations. Some pioneering works on multiplex lexical networks (Stella et al., 2017; Stella et al., 2018) also contain detailed supplementary information about the mathematics of multilayer networks and their relevance for psychologists interested in investigating the mental lexicon. More mathematically inclined psychologists would benefit from reading the theoretical formalism of multilayer networks, in general, described by pioneering papers in physics (Battiston et al., 2014; De Domenico et al., 2015; De Domenico et al., 2013; Santoro & Nicosia, 2020).

In addition to theoretical foundational papers, the physics community has also developed several ready-to-use tools for investigating multilayer networks. MuxViz, developed mostly by De Domenico et al. represents a powerful library for investigating the structure of multilayer networks (De Domenico, 2022), with an easy-to-use graphical interface and out-of-the-box data visualizations. MuxViz can also visualize small multilayer networks and detect potential overlaps between connections across different layers. A recent book (De Domenico, 2022) reports several tutorials about how to produce cutting-edge visualizations on multilayer networks. The R packages *igraph *and network provide more advanced data analysis and visualization options but through a steeper learning curve. Python users might rather resort to other valid alternatives, such as the Py3plex library, which performs multilayer network visualizations and analyses (Škrlj et al., 2019), or the packages *networkx* and *igraph* (available also in Python). Finally, the recent work by Aleta and Moreno (2019) represents a valuable tutorial paper for approaching multilayer networks and getting acquainted with their formalism for the first time.

## Conclusions

Cognitive multilayer networks can map multiple types of cognitive information at once. Their quantitative framework can thus model how different types of associations might co-exist within the mental lexicon and influence cognitive processing. This review has highlighted several pioneering studies unearthing mechanisms of psychological phenomena that could not be observed in single-layer cognitive networks. The phenomena that were only unveiled by the combination of multiple layers of associative knowledge included: (i) multiplex viability as a booster of lexical search and processing in people with lower creativity, shielding words from degraded production in people with aphasia; (ii) multilayer community detection as a way to highlight thematic clusters of concepts shaped by psycholinguistic norms and linked by multilayer shortcuts; and (iii) layer-layer correlations as interactive mechanisms between phonological and semantic similarities in lexical processing. In addition to describing the novel quantitative perspectives where multilayer networks can shed light on knowledge representations in the mental lexicon and in potential brain/mind models, we have discussed key limitations and promising directions for future research. The formalism covered in this review thus opens the way to next-generation quantitative frameworks of cognition able to model multivariate psychological data.
